# Sequential Isolation of Persistent Left Superior Vena Cava and Right Superior Vena Cava Using Pulsed-field Ablation with a Pentaspline Catheter for Recurrent Persistent Atrial Fibrillation

**DOI:** 10.19102/icrm.2024.15092

**Published:** 2024-09-15

**Authors:** Amulya Gupta, Murtaza Sundhu, Madhu Reddy, Seth H. Sheldon, Amit Noheria

**Affiliations:** 1 Department of Cardiovascular Medicine, The University of Kansas Medical Center, Kansas City, KS, USA

**Keywords:** Persistent atrial fibrillation, persistent left superior vena cava, pulsed-field ablation

## Abstract

Pulsed-field ablation (PFA) is a novel technology for atrial fibrillation (AF) ablation that can deliver energy precisely with a lower risk of damage to the surrounding organs. Persistent left superior vena cava (PLSVC) is a congenital variant that can act as a driver of AF, and its isolation may be required in recurrent persistent AF. We describe a case where PFA was used for isolation of the right superior vena cava, PLSVC, and posterior wall of the left atrium.

## Introduction

Pulsed-field ablation (PFA) is a novel, catheter-based, non-thermal technique for treating drug-refractory atrial fibrillation (AF). It uses electroporation to increase the permeability of cell membranes and induce cardiac cell death via apoptosis.^1^ Boston Scientific’s (Marlborough, MA, USA) FARAPULSE™ PFA system received the European CE mark in 2021 and subsequent U.S. Food and Drug Administration approval in January 2024, marking its recent commercial availability in the United States.^2,3^

Different tissues have different thresholds for injury from PFA. As cardiomyocytes have the lowest threshold for injury, it is believed that this technology electively targets cardiomyocytes, sparing the surrounding tissues, including the esophagus, the phrenic nerve, and the pericardium, potentially leading to reduced complications.^4^ A multicenter, single-blinded, randomized, non-inferiority trial comparing PFA to conventional thermal ablation demonstrated comparable efficacy and safety profiles for both treatments.^5^

Although there have been few head-to-head studies comparing PFA to other catheter ablation methods, a 2023 meta-analysis compared data from PFA with previously published data for other methods.^6^ Although not validated using statistical tests, PFA had a lower recurrence rate, higher rate of durable pulmonary vein isolation, and comparable complication rates.

In a recent meta-analysis comparing PFA with cryoablation, a statistically significant reduction in phrenic nerve palsy incidence following PFA was observed. However, despite similar overall complication rates between the modalities, PFA carried an increased risk of pericardial tamponade.^7^

Case reports regarding the use of PFA for right superior vena cava (RSVC) isolation and persistent left superior vena cava (PLSVC) individually have been reported in the literature.^8–12^ We present the first report of simultaneous isolation of the RSVC and PLSVC using PFA.

## Case presentation

**Figure 1:**
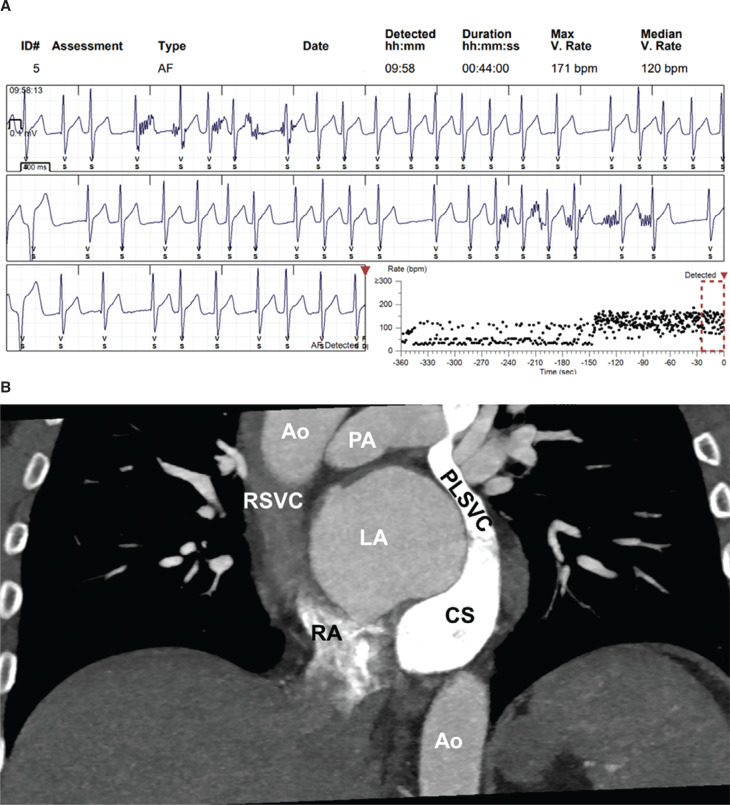
**A:** Implantable cardiac monitor recording of an episode of atrial fibrillation. **B:** Cardiac computed tomography angiography oblique view showing the patient’s persistent left superior vena cava and right superior vena cava. *Abbreviations:* AF, atrial fibrillation; Ao, aorta; CS, coronary sinus; LA, left atrium; PA, pumonary artery; PLSVC, persistent left superior vena cava; RA, right atrium; RSVC, right superior vena cava.

A 46-year-old man was referred to us with a history of bicuspid aortic valve, bioprosthetic aortic valve replacement, and recurrent persistent AF. He had had four prior catheter ablations for atrial arrhythmias, with the most recent procedure occurring 3 years ago. The procedure notes for his past ablations were not available. He was taking dabigatran, metoprolol, and dronedarone. He had an implantable cardiac monitor in situ. He continued to have AF recurrences with rapid ventricular response requiring three electrical cardioversions over the preceding 1 year **(Figure 1A)**. He was thus planned for another ablation procedure.

We conducted a pre-procedural chest computed tomography (CT) scan, revealing a normal left atrium (LA) and pulmonary veins, with no evidence of left atrial appendage thrombus. Additionally, an incidental finding of PLSVC draining into a dilated coronary sinus (CS) was noted, alongside the presence of an RSVC **(Figure 1B)**.

The procedure was performed under general endotracheal anesthesia. Bilateral femoral venous access was obtained, a 20-pole 2–10–2-mm spacing catheter was placed in the CS and right atrium, and a His-bundle recording catheter was placed. An intracardiac echocardiogram catheter was also inserted **(Video 1).** We used the Octaray™ catheter with Carto™ (Biosense Webster, Diamond Bar, CA, USA) to map the RSVC, right atrium, CS, and PLSVC in sinus rhythm. The activation map showed the sinus node exit site in the high right atrium. Large-voltage electrograms were recorded in the RSVC and PLSVC. There was no evidence of prior cavotricuspid isthmus, RSVC, CS, or PLSVC ablation. We proceeded with a single transseptal puncture using the VersaCross^®^ transseptal system (Baylis Medical, Montréal, Canada). We then placed the Octaray™ catheter in the LA and mapped it during sinus rhythm. This showed isolation of the bilateral pulmonary veins with normal-voltage bipolar electrograms in the rest of the LA, including the posterior wall.

The patient went into AF during catheter movement in the posterior wall, and a decision was made to isolate the posterior wall. The FARAWAVE™ (pentaspline) 31-mm PFA catheter was inserted in the LA. Using the flower configuration, overlapping lesions were created across the posterior wall from left to right with >50% overlap. Overall, 25 PFA trains were delivered. Post-ablation mapping with Octaray™ showed isolation of the posterior wall.

**Figure 2:**
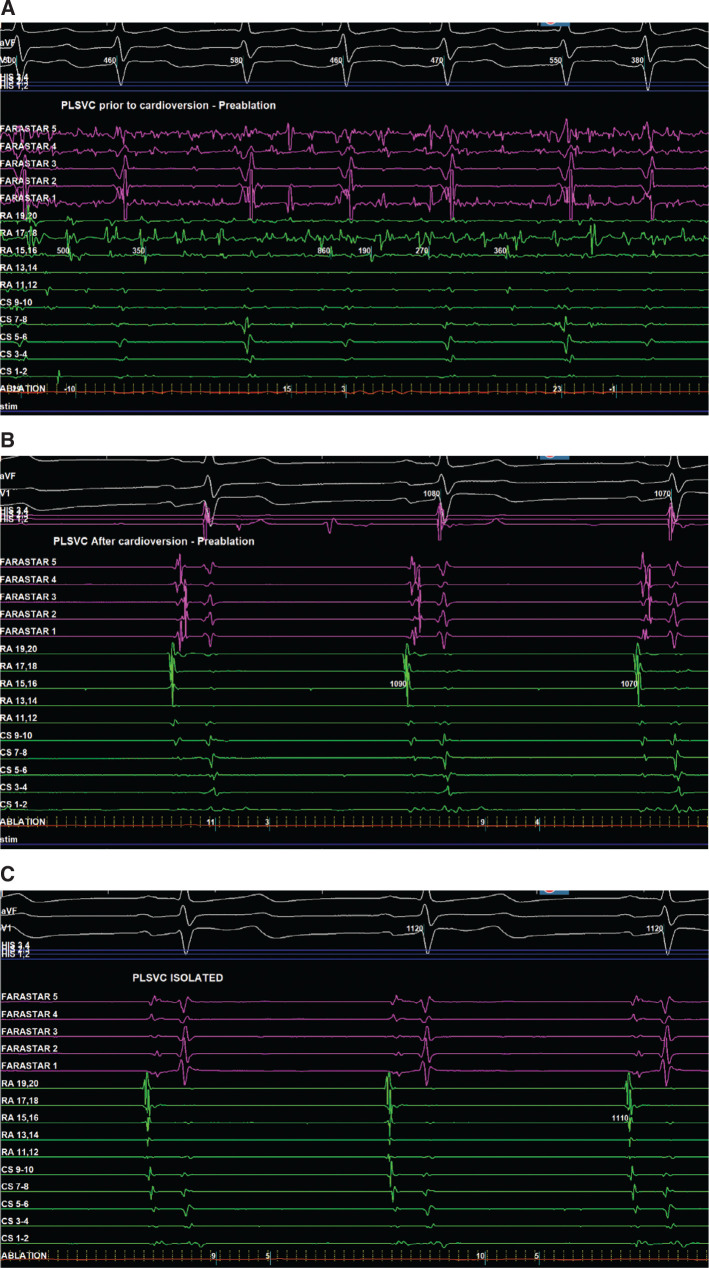
Electrograms recording in the persistent left superior vena cava (FARASTAR) and coronary sinus during atrial fibrillation **(A)**, after electrical cardioversion but before pulsed-field ablation **(B)**, and after pulsed-field ablation **(C)**, respectively. *Abbreviation:* PLSVC, persistent left superior vena cava.

**Figure 3:**
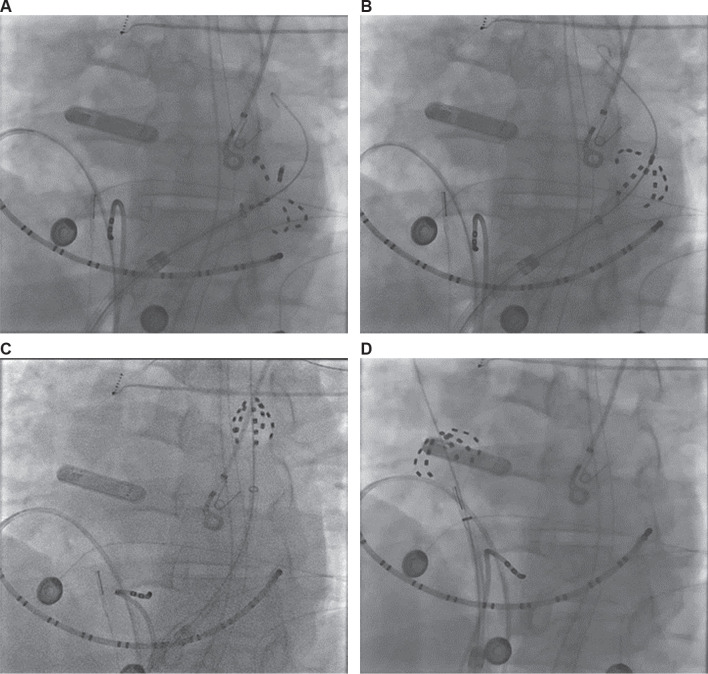
Left anterior oblique projection fluoroscopies of pulsed-field ablation of **(A)** the coronary sinus in flower, **(B)** the coronary sinus in basket, **(C)** a persistent left superior vena cava in olive, and **(D)** the right superior vena cava in basket configurations, respectively.

We removed the ablation catheter from the LA, and the PLSVC was cannulated. Cardioversion to sinus rhythm using 200 J of synchronized energy was completed so that the sinus node exit could be mapped and sinus node function monitored for RSVC isolation **(Figure 2)**. The Octaray™ catheter was inserted in the PLSVC and the Faradrive™ sheath was advanced over it into the CS. We transposed the coronary arteries from the cardiac CT scan on the mapping system, finding that the left circumflex artery was in proximity to the CS. To avoid vasospasm of the left circumflex artery with PFA application in the CS, we used 2 mg of nitroglycerin administered in the central venous system with 200 μg of phenylephrine pretreatment and waited for 1 min before completing PFA applications for 2 min. PFA applications were done in the “flower” and “basket” configurations in the mid-CS **(Figures 3A** and **3B)**. At the end of the 2 min, a repeat dose of 2 mg of nitroglycerin was given, and applications were completed for another 2 min. The patient’s blood pressure was supported while performing applications in the slightly distal portion of the CS until the PLSVC ostium. Lesions were created in smaller “basket” and “olive” configurations in the distal CS **(Figure 3C)**. A total of 22 PFA trains were delivered in the CS.

**Figure 4:**
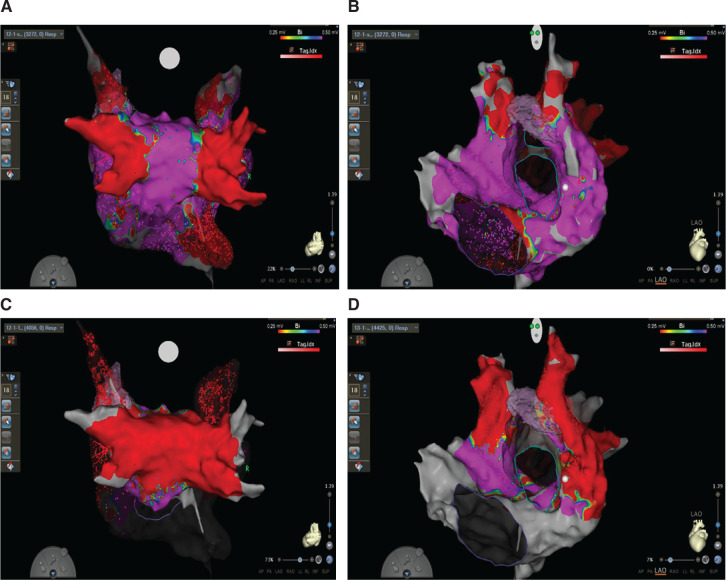
Electroanatomic map of pre-ablation **(A, B)** and post-ablation **(C, D)** bipolar voltage of a persistent left superior vena cava and right superior vena cava in the posteroanterior **(A, C)** and left anterior oblique projections **(B, D)**.

We then inserted the FARAWAVE™ catheter into the high right atrium. We recorded the local activation during sinus rhythm to ensure that, as the catheter was advanced to the SVC ostium, the local activation shifted later. This was also compared to the previously collected sinus rhythm activation map to ensure that we physically away from the sinus node exit site. Two milligrams of nitroglycerin was used to avoid spasm of the sinoatrial nodal artery. Four PFA trains were delivered in a flower configuration outside the SVC ostium, and an additional four trains in an olive configuration were delivered just inside the SVC ostium **(Figure 3D)**. We did not see any dropped sinus beats or slowing of the sinus rate. A final map of the right atrium and PLSVC was completed to confirm isolation of the RSVC and PLSVC **(Figure 4)**.

After the ablation procedure, the patient first received 5 μg/min and then 10 μg/min of isoproterenol intravenously. Burst pacing was performed, reducing the pacing interval to 200 ms from the high right atrium and proximal CS. Notably, there was no induction of AF or atrial flutter.

A total of 55 PFA applications, including 25 on the posterior wall, 22 in the CS, and 8 for the RSVC, were delivered. The patient tolerated the procedure well and was discharged home the next day uneventfully. Anticoagulation with dabigatran and anti-arrhythmic therapy with dronedarone were continued.

## Discussion

PFA is gaining popularity as a method for AF ablation. As the technology is relatively new, long-term outcomes are yet to be evaluated. In our case, the patient had recurrent persistent AF despite pulmonary vein isolation and was later found to have PLSVC on CT imaging. Previously, PLSVC has been implicated as a possible driver of AF.^13^ Additionally, the posterior LA, the RSVC, and the CS have been shown to drive AF in some patients.^14^ Therefore, additional isolation of these structures was pursued in an attempt to suppress AF in this patient with four prior ablation procedures. While studies have shown the efficacy and safety of SVC isolation for conventional catheter-based methods, such methods are yet to be validated for PFA.^15^

Coronary vasospasm is an important side effect observed with PFA application near coronary arteries.^16^ Nevertheless, pretreatment with high-dose nitroglycerin boluses can be an effective strategy in preventing severe coronary vasospasms.^17^

The application of thermal methods for posterior wall isolation has been hindered by challenges in delivering sufficient energy while minimizing risks to surrounding structures, notably the esophagus.^18^ Complications stemming from esophageal injury include erosion, ulceration, and severe atrioesophageal fistula, associated with a considerable mortality rate. However, the tissue specificity of PFA largely mitigates these concerns, enabling appropriate energy delivery and the potential for establishing durable posterior wall isolation.

## Conclusion

Our case demonstrates that, in patients with extrapulmonary vein-triggered AF in the presence of PLSVC, simultaneous isolation of PLSVC and RSVC can be done safely using the pentaspline PFA catheter.

## Figures and Tables

**Video 1:** Intracardiac Echocardiogram (ICE).
